# Water, Sanitation, and Hygiene for Everyone Intervention Study: Protocol for a Controlled Before-and-After Trial

**DOI:** 10.2196/68280

**Published:** 2025-05-15

**Authors:** Kondwani Chidziwisano, Mindy Panulo, Clara MacLeod, Marcella Vigneri, Blessings White, Joseph Wells, Ian Ross, Tracy Morse, Robert Dreibelbis

**Affiliations:** 1 Centre for Water, Sanitation, Health and Appropriate Technology Development (WASHTED) Malawi University of Business and Applied Sciences Blantyre Malawi; 2 Department of Civil and Environmental Engineering University of Strathclyde Glasgow United Kingdom; 3 Department of Public and Environmental Health Malawi University of Business and Applied Sciences Blantyre Malawi; 4 Department of Disease Control London School of Hygiene & Tropical Medicine London United Kingdom

**Keywords:** community-led total sanitation, care group model, WASH for everyone, Malawi, sanitation intervention trial

## Abstract

**Background:**

Community-based behavior change interventions are a common approach to Water, Sanitation, and Hygiene (WASH). Yet, published evaluations of how these interventions work in district-wide approaches are rare.

**Objective:**

This study reports the baseline characteristics and study design for a trial assessing the effectiveness of a district-level Community-led Total Sanitation (CLTS) intervention compared to the additional integration of local care groups (CG) on sanitation coverage and use and hygiene behaviors in Chiradzulu District, Malawi.

**Methods:**

This study is a controlled before-and-after trial with 2 treatment arms and a control group. Clusters are rural villages in 3 traditional authorities (TAs). One arm will receive CLTS and the CG model (CLTS+CG group), one arm CLTS only (CLTS group), and one group will serve as the control. The trial is part of the wider WASH for Everyone (W4E) project, led by World Vision Malawi that aims to expand access to WASH services across the entire district by 2025. Study participants were selected from the 3 TAs. Systematic sampling procedures were used to select 20 households per cluster with a total of 1400 households at both baseline and end line. The primary outcome is sanitation coverage. Secondary outcome measures include sanitation use, safe disposal of child feces, observed handwashing facility, and Sanitation-related Quality of Life Index (SanQoL-5).

**Results:**

The baseline observations indicate a balanced distribution of potential demographic confounders in the trial arms with a slight variation on some WASH proxy measures. We noted the low coverage of handwashing facilities with soap and water in all 3 arms: 8% in the CLTS group, 4% in the CLTS+CG group, and 4% in the control group. There was a marginal variation in handwashing practices among the study arms with 3% of individuals handwashing with soap and water in the CLTS group, 5% in the CLTS+CG group, and 2% in the control group. Sanitation coverage also varied among the study arms at baseline as 83% of households had access to unimproved sanitation in the CLTS group, 70% in the CLTS+CG group, and 81% in the control group.

**Conclusions:**

Results from this trial will provide evidence on whether the CLTS+CG approach is effective at improving sanitation and hygiene practices in the W4E program area compared to CLTS alone and no intervention, as well as inform implementing partners on future interventions in Chiradzulu District, Malawi. The results are expected to be published in 2025.

**Trial Registration:**

ClinicalTrials.gov NCT05808218; https://clinicaltrials.gov/study/NCT05808218

**International Registered Report Identifier (IRRID):**

RR1-10.2196/68280

## Introduction

Globally, it is estimated that 3.6 billion people lack access to basic sanitation services and 494 million people practice open defecation (OD), with the highest rates of OD in Sub-Saharan Africa [1]. Consequences of OD include fecal contamination of drinking water sources and food, which contributes to a high burden of diarrheal diseases and child stunting, adversely impacting health and socioeconomic development [2,3]. Further, OD and inadequate sanitation disproportionately affect the safety and dignity of women, girls, and marginalized groups [3-6], as well as other aspects of quality of life [7]. Efforts by governments and other sanitation stakeholders to eliminate OD, such as the provision of subsidized latrines to households combined with hygiene and health education programs, have failed to make adequate sustained progress [8,9]. Behavior-centered interventions have been associated with improved uptake of sanitation interventions, but more evidence is needed to assess their impact on behavioral outcomes when implemented in combination with one another.

Our study focuses on 2 specific community-led interventions widely used in the Water, Sanitation, and Hygiene (WASH) sector. The first is Community-Led Total Sanitation (CLTS), an approach to sanitation behavior- change centered on community-wide behavior change and community self-enforcement in rural settings [9]. Introduced in Bangladesh in 2009 and now adopted globally, the major goal of CLTS is to mobilize communities to construct and use latrines to end OD [9]. CLTS uses 3 phases to leverage social and emotional drivers to “trigger” a change in people’s mindsets towards OD [10]. Evidence on the efficacy of CLTS is mixed. Certain studies highlight that CLTS only generates significant short-term impact for reducing OD through increased latrine coverage and use [9,11-14]. CLTS implementation factors, such as triggering session attendance, the number of supportive community leaders, participants’ anticipation to receive an incentive, and the number of follow-up visits, have been reported to significantly influence latrine coverage [15]. The second is the care group (CG) model which relies on a multiplier effect to reach a high number of households in a community at a low cost through the development of a supportive network of peer-to-peer counseling [16]. The CG model is a well-tested program for the delivery of health interventions in rural communities, historically focusing on maternal and child health [16-19]. A total of 23 nongovernmental organizations (NGOs) have implemented the CG model across 27 countries, including Malawi [17,19]. Studies have documented the effectiveness of the CG model in increasing coverage of child survival interventions and reducing under-five mortality [18-20]. However, despite their clear alignment, studies of the effectiveness of CGs as they relate to WASH interventions are limited.

In Malawi, communities struggle to sustain 100% latrine coverage after attainment of Open Defecation Free (ODF) status [21]. This has been attributed to a number of factors, including the lack of involvement of marginalized and disadvantaged people, the use of low-quality building materials, lack of technical support, and improper program implementation [14,22]. To achieve high and sustained latrine coverage and behavior change, it is essential to address all physical and contextual factors that directly relate to long-term CLTS success. The Government of Malawi adopted CLTS as one of its official approaches to sanitation in 2008 [23]. The Government formally adopted the CG model in 2011 as an operational framework for the Scaling Up Nutrition (SUN) Strategy [24]. However, little is known about how effective the CG model can be in promoting wider community health benefits, such as improved sanitation. Models to promote sustained reductions in open defecation and improved sanitation outcomes through a combination of behavior-centered intervention need to be tested and adopted to support long-term positive health outcomes.

This study aims to assess the effectiveness of CLTS combined with the CG model on sanitation coverage and use and hygiene behaviors in Chiradzulu District, in rural Malawi, compared to CLTS alone or with no intervention. The study objectives are to assess: (1) how the 2 interventions compare with one another for improving sanitation coverage and use, and (2) whether the 2 interventions are individually more effective than no intervention at all.

## Methods

### Study Setting and Population

**Figure 1:**
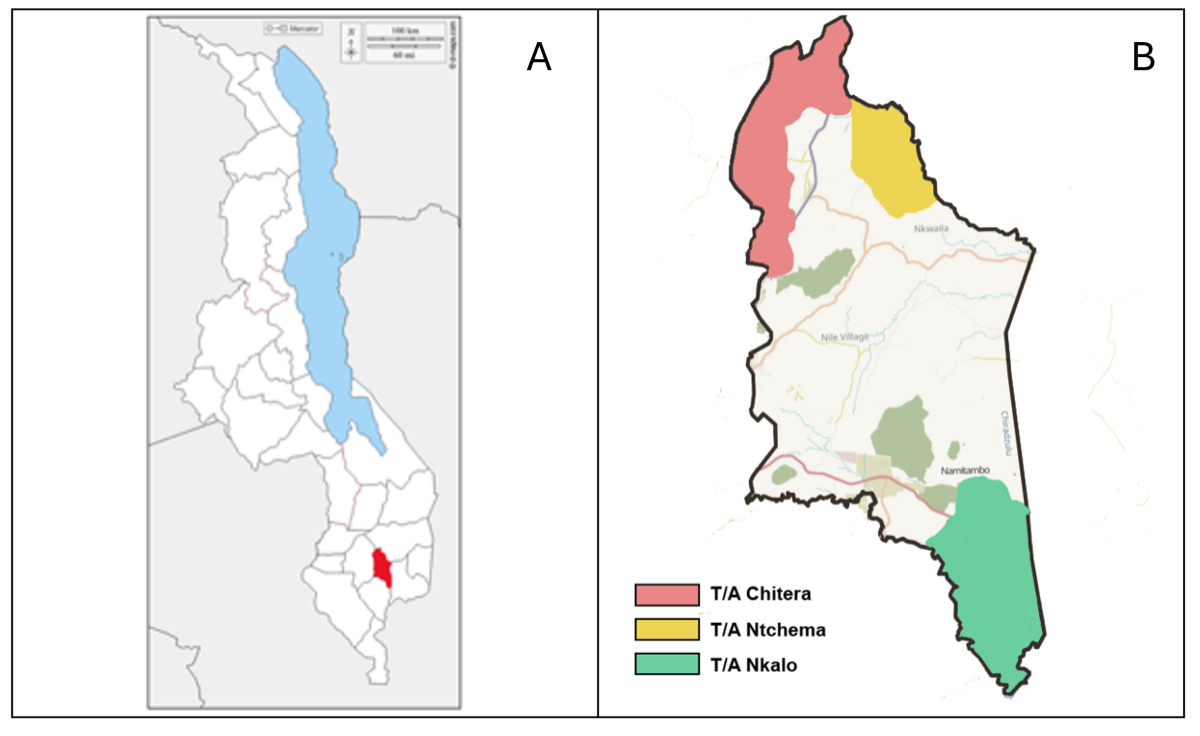
(A) Map of Malawi with Chiradzulu District in red, and (B) Map of Chiradzulu District with W4E study areas with TA Chitera (CLTS+CG group) in red, TA Ntchema (CLTS only) in yellow, and TA Nkalo (control) in green. CG: care group; CLTS: community-led total sanitation.

The study is implemented in Chiradzulu District, Malawi (Figure 1). Chiradzulu District is situated in the southern region of Malawi and is subdivided into 10 administrative regions, or traditional authorities (TA). The Malawi 2015-2016 Demographic and Health Survey (DHS) indicated that 52% of the population has access to improved sanitation. According to the National Statistical Office, in 2019, 93% and 10% of the households in Chiradzulu District had access to safe water and improved sanitation facilities, respectively. OD rates in Malawi and Chiradzulu District are 6% and 7%, respectively [25]. Given this low level of coverage, Chiradzulu District is the target of a 3-year (2021-2024) district-wide water, sanitation, and hygiene program, known as WASH for Everyone (W4E), implemented by World Vision and Water for People, alongside which this trial is embedded.

### Study Design

**Figure 2:**
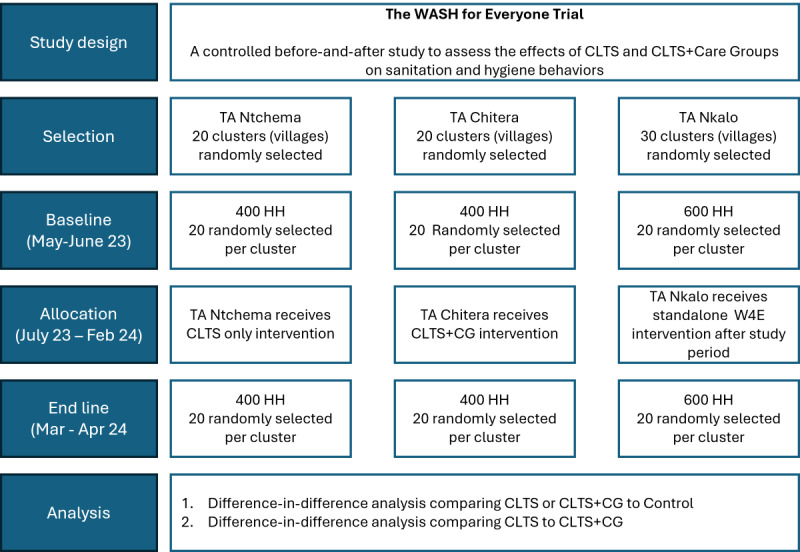
Study CONSORT (Consolidated Standards of Reporting Trials) diagram for a controlled before-and-after trial to assess the effectiveness of two interventions (CLTS only and CLTS+CG) on rural sanitation coverage and use in Chiradzulu District, Malawi. CG: care group; CLTS: community-led total sanitation.

The study design is a controlled before-and-after (CBA) trial with 2 treatment arms (each with 20 villages or clusters) and a control group (30 villages; Figure 2; Multimedia Appendix 1). CBA intervention designs are a nonrandomized approach used to evaluate the impact of interventions [26]. The advantages and disadvantages of the CBA study designs, also known as nonrandomized cluster-controlled trials, have been discussed elsewhere [27]. In this study, TAs are the unit of intervention assignment and villages (clusters) are the unit of analysis. [28] In our study, we selected 3 TAs (Figure 1) in Chiradzulu District, Malawi. TAs are 4th-level administrative units, with an average population of 34,000 in Chiradzulu [29]. Details on the program intervention are described below. For our trial, we selected 2 out of the 5 TAs scheduled to receive the full W4E intervention during the second year of program implementation to align with our implementation. We selected the 2 TAs with the closest match in population and estimated sanitation coverage from previous surveys. In discussion with program partners, one TA was assigned to receive the CLTS intervention, and one TA was assigned to receive the CLTS+Care Group intervention. We selected a third TA from the 3 TAs scheduled to receive the WASH for Everyone intervention in the third year of program activities as a control group for our study (Figure 2). The control TA was selected based on generally similar population and sanitation coverage measures and lack of contiguous borders with the 2 intervention TAs. TAs are the unit of intervention assignment for our study. Within each TA, villages serve as the specific study clusters which are the primary sampling units for data collection.

### Sample Size

Sample size calculations were based on the minimum detectable effect (MDE) to assess changes in sanitation coverage, the primary outcome measure, at the project end line. Data for all parameters used in sample size calculations were drawn from the baseline survey conducted by World Vision before the implementation of the W4E project between January and April 2021. For the MDE calculation, we assumed a 2-tailed test with a significance level of 0.05 and a power of 0.8. We assumed a conservative intracluster coefficient of 0.1 [30]. As baseline data indicates that the average percentage of households with at least unimproved sanitation coverage across Chiradzulu District was 67%, power calculations assumed a total enrollment of 20 intervention clusters in each TA receiving interventions and 30 clusters selected from the control arm of the study. Assuming an average of 20 households surveyed per selected cluster results in an MDE of 0.136 or an average difference of 13.6 percentage points in sanitation coverage (relative change in coverage of 19%) between either arm of intervention clusters and the control cluster. Therefore, the study aims to recruit a total of 1400 households (CLTS+CG: n=400; CLTS only: n=400; Control: n=600; Figure 2) at each data collection round.

### Description of the Intervention

Households in TA Ntchema (CLTS only) will receive standard CLTS intervention plus government-delivered hygiene promotion. We refer to this arm as the CLTS group. Households in TA Chitera (CLTS+CG group) will receive the same combination of interventions as the CLTS group in addition to community-based CGs. We refer to this as the CLTS+CG group.

CLTS is the main sanitation intervention component for W4E and is implemented in line with the Malawi National Sanitation and Hygiene Strategy [29]. As part of W4E, relevant district technical officers and community leaders (also known as Natural Leaders) are responsible for implementing CLTS. Trained CLTS facilitators will conduct the triggering sessions that include participatory activities, such as the walk of shame, shit calculation, and community sanitation mapping. The purpose of these activities is to trigger behavioral emotions, such as shame and disgust so that community members understand the consequences of open defecation. If successfully implemented, triggering sessions have the potential to stimulate community members to stop open defecation and adopt improved sanitation practices, including the construction and effective use of latrine facilities. In addition to sanitation promotion, the project partners delivered hygiene promotion campaigns using pre-established sanitation and hygiene messages from the Ministry of Health in marketplaces and villages. This was achieved through 4 separate campaigns, each lasting 5 days, using a mobile van for message dissemination and distributing information leaflets.

In TA Chitera, in addition to the activities outlined above, households will receive visits from local CGs. CG leaders and cluster leaders support intervention delivery and local triggering sessions, specifically by facilitating CG meetings with CG households and conducting household follow-up visits. CGs are intended to extend the reach of CLTS behavior change messaging, providing additional points of contact with program households. CGs also participate in posttriggering follow-up visits to households to assess sanitation coverage and use.

In line with CLTS values, the W4E project does not intend to provide any latrine construction or hygiene facility materials or financial subsidies to households. It is the responsibility of the household owners to support themselves throughout the latrine and hygiene facility construction process.

### Selection of Clusters and Households

The primary sampling units (clusters) for the study are clusters (also called villages) and represent the units of W4E delivery for CLTS. Communities from the 3 participating TAs were randomly selected from a list of communities obtained from the Chiradzulu District Health Office. Inclusion criteria for the selected communities include that the community is in one of the 3 selected TAs that are part of the study area and have not yet received any exposure to the W4E project. Communities that were exposed to CLTS-related activities in the past 12 months and are outside the area of the 3 selected TAs are excluded from the study.

In early 2023, a list of all clusters in each study TA and their associated number of households was provided by the District Health Office. TA-specific median village size was calculated, and villages were categorized as either above or below the TA-specific median. Villages in each TA-specific above and below median list were ranked ordered according to a random number generated in Microsoft Excel and villages were enrolled sequentially until the necessary number of villages were enrolled. If a cluster could not be located or if the village chief did not provide permission for data collection, the next cluster on the rank order list was enrolled.

Households are the secondary sampling unit and individuals living in households in the study area are the primary study population. Twenty communities will be selected from TA Chitera (CLTS+CG), 20 from TA Ntchema (CLTS), and 30 from TA Nkalo (control). Among selected communities, a systematic sampling procedure (selecting every kth household) was used to select 20 households from each cluster included in the study. For each cluster, the population of households obtained from the District Health Office was divided by 20 (cluster sample size) to find the sampling interval (kth number). Then, the kth household was selected and included in the survey from any starting household chosen by an enumerator within the cluster.

### Data Collection

A baseline survey was conducted in all 3 TAs between May and June 2023 before intervention implementation in TA Chitera (CLTS+CG group) and TA Ntchema (CLTS group). An end-line survey was conducted in all 3 TAs between March and April 2024. TA Nkalo (control group) received the CLTS intervention after the end-line survey. A structured questionnaire with closed-ended questions with precoded responses on mobile devices on the KOBO collect platform (KoboToolbox) was used to collect data on household membership, wealth index, sanitation and hygiene facilities, and child health (Multimedia Appendix 2). Further, the enumerators conducted spot checks and recorded hygiene proxy measures, such as the presence and state of a latrine, presence, location, and type of handwashing facility (including the availability of soap and water). The interviews were conducted in Chichewa, the local language of Chiradzulu District. Chichewa-speaking enumerators with extensive training and expertise administered the structured questionnaire.

To confirm the availability of WASH infrastructure and hygiene practices, we conducted structured observations in 350 selected households (ie, 100 households from each intervention arm and 150 households from the control group) among the recruited 1400 households. A line listing of the 1400 households was created and a Microsoft Excel random generation number was run to identify the 350 households for observations. Specifically, the observations were intended to capture practices pertaining to the presence of latrine and handwashing facility with soap, handwashing practice at critical times (ie, before eating, before food preparation, after changing the child's nappy, and after latrine use), and child feces disposal. One observer was placed at each selected household. As behaviors of interest mostly occur in the morning hours to noon [31], observations lasted 4 hours from 8 AM to noon. The observer first conducted the structured observations after which he or she proceeded to administer the structured questionnaire.

### Data Management

Data collected using mobile devices was uploaded directly on the web to KOBO daily. Only the PI, coinvestigators, and study personnel with authorized access have access to web-based data. At the end of the data collection, the full dataset was uploaded into STATA (version 18; StataCorp) for analysis. Although no identifiable information was recorded during the surveys, study personnel will review the final database and permanently delete any identifiable information inadvertently collected during surveys. All data is stored on encrypted, password-protected servers.

### Study Outcomes

The primary outcome is sanitation coverage. Secondary outcomes include basic sanitation coverage, sanitation use, sanitation-related quality of life, latrine quality, presence of a handwashing facility, handwashing behavior, and safe disposal of child feces (Multimedia Appendix 3).

### Analysis

Statistical analysis will be carried out at the individual or household levels with appropriate adjustments for clustering within villages (and within households for individual-level outcomes). Data will be analyzed according to the TA and intervention assignment irrespective of whether the intervention was taken up fully, partly, or not at all. Since this is an effective study, only an intention-to-treat dataset will be maintained.

The primary estimates of the effectiveness of the intervention on primary and secondary outcomes will be based on a difference-in-difference analysis and models adjusted for design variables alone (TA, village population above or below TA-specific median); a fixed effect for village size (above or below the median) will be included in the model. All models will include a dummy variable for the treatment arm (ie, CLTS, CLTS+CG, or control) and data collection round (baseline and end line) as well as the interaction term of those 2 variables.

To account for the clustering of observations, hierarchical mixed-effects models will be used for all analyses. For household-level outcomes or outcomes where there is only one respondent per household, mixed effects models will account for clustering by including a random effect at the village level. For outcomes with multiple respondents or data points per household (specifically sanitation use, and hand washing behavior), an additional random effect will be added at the household level.

All analysis of primary and secondary outcomes will be based on the difference-in-difference approach. The regression coefficient of the interaction term between the treatment arm and the data collection round will be used as the primary effect measure. For binary outcomes measure, we will use melogit with robust SEs and regression coefficients exponentiated to estimate an odds ratio (OR). For continuous outcomes, we will use meglm, with identity link and Gaussian family.

The results will be presented with 95% CIs. Analyses will report on ORs and 95% CIs of each treatment arm compared to the control as well as the CLTS alone compared to CLTS+CG. Secondary outcomes include the following:

1. Sanitation use: measured as an individual-level binary indicator based on the reported location last time each member of the household used a latrine.
2. Safe disposal of child feces: will be analyzed as a mixed effect model (household, village, and cluster) of observed household disposal sites of last defecation for all children under the age of 5 years.
3. Basic sanitation coverage: this is a household-level indicator based on the presence of an observed sanitation facility that meets Joint Monitoring Programme (JMP) criteria for basic sanitation facilities, specifically an improved sanitation type.
4. Sanitation-related Quality of Life (SanQoL-5): SanQoL-5 index is an individual-level variable reported by only one participant per household. It is a continuous variable (0-1) based on the weighted score of responses to 5 questions on a 3-level frequency scale [32].
5. Latrine quality: latrine quality is a composite index based on observed and reported characteristics of household latrines [8]. Specific binary indicators will be combined using principal components analysis (PCA) and iteratively refined using standard approaches to index development. Outcomes will be analyzed as a continuous variable based on the PCA score.
6. Basic handwashing facility: the presence of handwashing where both soap and water are available is a household-level binary variable. This will be analyzed in two ways: (1) both observed and reported handwashing facility (HWF) with both soap and water available at the time of data collection and (2) observed HWF with soap and water.
7. Handwashing behavior: A binary outcome measure based on structured observation data. Structured observation data will be used to identify all predefined hand hygiene opportunities (eg, before food preparation, before eating, before feeding a child, after using a latrine, after cleaning a child, and after being in contact with an animal) and associated hand hygiene (0=no hand hygiene or hand hygiene with water only; 1=hands washed with soap). The analysis will be conducted at the event level with adjustment for repeated observations within the same household.

### Adjustment for Covariates

Results will present 2 sets of outcome measures. First, we will report on all outcome measures adjusted for design variables (village above or below TA-specific median) and models also adjusted for a priori-defined covariates and design variables. We will explore differences between the design-adjusted and covariate-adjusted models but will consider the covariate-adjusted models as the primary effect estimates.

Covariates have been selected based on hypothesized relationships that could confound the relationship between intervention exposure and primary and secondary outcome measures.

At the respondent level, gender and primary education (less than vs completed primary) will be included. The following covariates will be included at household-level: household size, household economic status based on PCA of household assets, any member of the household experiencing a disability as defined by the Washington Group (any functional disability for a member of the household greater than 2 on the functional disability assessment) and household reports a water source that is located on site or on plot [33]. At the community level, we will look at the village having an improved road as reported by the Village Chief at the time of baseline enrollment. The improved road is used as a proxy measure of village accessibility to both the intervention and markets.

### Missing Data

It is likely that some missing outcome data will be encountered, especially for individual-level self-reported outcomes. The patterns of missingness of variables will be tabulated to describe and compare the extent of missingness of any affected variable between study arms. No adjustment will be made for missing data.

### Outliers

Unusual values and potential outliers will be flagged and queried. Unlikely values will be dropped and treated as missing data in the main analysis. A sensitivity analysis will be conducted which includes potential outliers (but not unlikely values).

### Multiple Comparisons

The number of primary outcomes that will be tested for significant differences between arms is small; thus, no formal adjustment for multiple comparisons will be made.

### Ethical Considerations

The study protocol, which includes data collection tools, participant information sheets, and consent forms, has been approved by the National Commission for Science and Technology (P01/23/718) in Malawi and the London School of Hygiene and Tropical Medicine (28249). Further, consent was obtained from the Chiradzulu District Council and community leaders. Informed written consent was obtained from all study participants recruited into the study before data collection. The trial was registered in ClinicalTrials.gov (NCT05808218).

## Results

Most baseline characteristics, such as gender, education, marital status, presence of children under 5, and wealth quintile, were balanced across the 3 study groups (Table 1). A total of 400 respondents were sampled from TA Chitera (CLTS+CG group), 400 from TA Ntchema (CLTS group), and 600 from TA Nkalo (control group). Across the 3 TAs, most survey respondents were female (83%), had at least some primary education (67%), and were married (68%). The median household size was 4 members. Approximately half (49%) of households had a child under the age of 5 years. Around 6% of households had at least one member living with a disability. The baseline results indicate some imbalance between trial arms on self-reported water treatment practices.

At baseline, 1094 out of 1400 (78%) households had an unimproved sanitation facility as defined by JMP (eg, a pit latrine without a slab). Specifically, most households (76%) had a pit or twin pit latrine without a slab and in a yard or plot (68%). The median number of households among those sharing one latrine was 3. A total of 5183 out of 5386 (96%) individuals reported using a sanitation facility for their last defecation event. The mean SanQoL is 0.62. Around 980 out of 1395 (70%) households used an improved water source as defined by JMP (98%; Table 2), with 1317 out of 1400 (94%) households using a borehole as their main water source. Around 25 minutes was the median round-trip time to collect water. A total of 787 out of 1400 (56%) households had access to a handwashing station, either observed or reported (Table 2). However, only 104 out of 1400 (7%) households had a handwashing facility with reported or observed soap and water available at the handwashing facility.

**Table 1:** Description of baseline respondent characteristics.

Variable			All participants (N=1400)		Control (TA Nkalo) (n=600)		CLTS+CG (TA Chitera) (n=400)		CLTS (TA Ntchema) (n=400)
Sex (female), n (%)			1167 (83)		510 (85)		317 (79)		340 (85)
Median age			40		39		39		40
**Education, n (%)**									
	None	107 (9)		44 (7)		30 (8)		33 (9)	
	Less than primary	43 (3)		25 (4)		9 (2)		9 (2)	
	Completed primary	940 (67)		405 (68)		246 (61)		289 (72)	
	Secondary	296 (21)		119 (20)		110 (27)		67 (17)	
	Vocational	6 (0)		2 (1)		3 (1)		1 (0)	
	University	8 (0)		5 (0)		2 (1)		1 (0)	
**Marital status, n (%)**									
	Single	85 (6)		33 (6)		33 (8)		19 (5)	
	Married	958 (68)		37 (67)		20 (71)		15 (69)	
	Separated or divorced	208 (15)		103 (17)		48 (12)		57 (13)	
	Widow	149 (11)		62 (10)		37 (9)		50 (13)	
Median household size, n			4		5		4		5
Presence of children under 5 years of age, n (%)			171/350 (49)		68/150 (45)		53/100 (53)		50/100 (50)
**Wealth quintile, n (%)**									
	1	281 (20)		115 (19)		80 (20)		86 (22)	
	2	279 (20)		114 (19)		71 (18)		94 (24)	
	3	280 (20)		135 (23)		72 (18)		73 (18)	
	4	280 (20)		123 (21)		81 (20)		76 (19)	
	5	280 (20)		113 (19)		96 (25)		71 (18)	
At least one household member living with a disability, n (%)			72 (6)		31 (6)		18 (5)		23 (6)

**Table 2:** Description of outcomes at baseline.

Characteristics		All participants	Control (TA Nkalo)	CLTS+CG (TA Chitera)	CLTS (TA Ntchema)
Improved water source (JMP^a^), n/n (%)		1364/1395 (98)	587/598 (98)	384/397 (97)	393/400 (98)
At least basic drinking water (JMP), n/n (%)		980/1395 (70)	466/598 (78)	253/397 (63)	261/400 (65)
Report treated water at least once a day, n/n (%)		876/1400 (63)	342/600 (57)	255/400 (64)	279/400 (70)
**Sanitation (MP), n/n (%)**					
	At least basic	97/1400 (7)	26/600 (4)	43/400 (11)	28/400 (7)
	Limited	149/1400 (11)	55/600 (9)	64/400 (16)	30/400 (8)
	Unimproved	1094/1400 (78)	483/600 (81)	278/400 (70)	333/400 (83)
	No facility	60/1400 (4)	36/600 (6)	15/400 (4)	9/400 (2)
Number of households sharing one latrine (among those sharing), median (IQR)		2 (2-3)	2 (2-3)	3 (2-3)	2 (2-3)
All members of the household (aged ≥5) reported using the latrine for last defecation, n/n (%)		5183/5386 (96)	2201/2338 (94)	1480/1520 (97)	1502/1528 (98)
SanQoL-5 index, mean (SD)		0.62 (0.25)	0.59 (0.26)	0.64 (0.26)	0.64 (0.24)
Reported safe child feces disposal, n/n (%)		641/715 (90)	263/291 (90)	184/209 (88)	194/215 (90)
**Handwashing facility available (reported or observed), n/n (%)**					
	Basic^b^	104/1400 (7)	59/600 (10)	15/400 (4)	30/400 (8)
	Limited^c^	683/1400 (49)	309/600 (52)	168/400 (42)	206/400 (52)
	No handwashing facility	613/1400 (44)	232/600 (39)	217/400 (54)	164/400 (41)

^a^Joint Monitoring Programme.

^b^Basic handwashing facility: handwashing facility with soap and water.

^c^Limited handwashing facility: handwashing facility with no soap or water.

A total of 1099 hand hygiene structured observations were conducted across the 3 TAs (Table 3). Specifically, 330 structured observations were conducted in TA Chitera (CLTS+CG), 295 in TA Ntchema (CLTS), and 474 in TA Nkalo (control). The median number of observed hand hygiene opportunities per household was 3. Most households did not practice hand hygiene or wash hands with water at specific hand hygiene junctures (eg, after using the toilet, before food preparation, before eating, before feeding a child, and after being in contact with an animal; Table 3). A total of 73 child feces disposal structured observations were conducted across the 3 TAs, with 26 in the CLTS+CG group, 18 in the CLTS, and 29 in the control. Overall, approximately half of the households safely disposed of child feces (Table 3).

**Table 3:** Hand hygiene and child feces disposal structured observations.

Characteristics			All participants (N=1099)		Control (TA Nkalo) (n=474)		CLTS+CG (TA Chitera) (n=330)		CLTS (TA Ntchema) (n=295)
**Hand hygiene, n (%)**									
	Handwashing with soap and water	36 (3)		12 (2)		17 (5)		7 (2)	
	Handwashing with water only	505 (46)		231 (49)		128 (39)		146 (50)	
	Handwashing with other materials (ie, ash)	2 (0)		0 (0)		1 (0)		1 (0)	
	No handwashing	556 (51)		231 (49)		184 (56)		141 (48)	
Safe child feces disposal^a^, n/n (%)			36/73 (49)		13/29 (45)		14/26 (54)		9/18 (50)

^a^Safe child feces disposal is defined as child latrine use or caregiver disposal of child feces into the latrine.

## Discussion

### Anticipated Findings

Given that CLTS alone is reportedly not effective at ensuring sustained uptake of sanitation and hygiene behaviors, we test the hypothesis that the CLTS+CG intervention will be more effective at improving sanitation coverage in a rural area of Malawi than the CLTS intervention alone, and also relative to the no intervention arm. Studies have shown the effectiveness of using the CG model in promoting community health interventions, mainly nutrition programs [19,34-37]. However, there is limited evidence on the impact of interventions integrating CLTS and the CG model to address this gap, we tested an intervention that integrates the CG model into the standard CLTS approach. The CG model is a community-level government-recognized approach that can potentially support the already existing informal natural leaders in the delivery of CLTS, both pre- and post-ODF status attainment. This could strengthen the sustainability of CLTS as CG members are trained to continuously monitor groups of households post-ODF attainment, whereas Natural Leaders only follow up with households until the community has achieved ODF status [9,11,38,39]. Results from this trial will provide evidence on the extent to which the CLTS+CG approach is effective at improving sanitation and hygiene practices in the W4E program area compared to CLTS alone and no intervention, as well as inform implementing partners on future interventions in Chiradzulu District, Malawi.

Our baseline results indicate that the study is conducted in a rural area where the literacy level is moderate and the quality of housing relatively poor. Some households had no latrines, while traditional unimproved latrines (eg, pit latrines without slab), which are prone to collapse during the rainy season, are common. Relatively high sanitation coverage in the communities indicates the short-term success of CLTS, however, other TAs in Chiradzulu District have reverted to OD after achieving ODF status. Thus, the district provided a suitable environment for this study to assess alternative approaches for promoting sustainable sanitation interventions.

Handwashing with soap is low in low- and middle-income countries, including Malawi [40-44]. Similar findings were observed in our baseline results. Access to easy-to-use and effective handwashing facilities was limited among the study population as most households did not have a dedicated handwashing facility. Observations at critical times indicated a challenge with soap availability for hand washing. As such, nearly half of the study participants did not use soap during handwashing.

The baseline results indicate some imbalance between trial arms on self-reported water treatment practices. Treatment of household water was more frequently reported among the control group compared to the treatment groups. A possible explanation for this imbalance could be that immediately before baseline data collection, other NGOs promoted the use of safe water through the drilling of boreholes and installation of chlorine dispensers in all the water points in the control communities. This included the installation of chlorine dispensers in both treatment groups, although a similar trend was not observed in responses. In terms of sanitation, we observed that the control arm had the highest coverage of latrines without a slab, while more latrines with a slab were observed in the CLTS group. We could not find an explanation for this difference and expect it to be by chance.

This study has several limitations. Structured observations were used to collect the data. This approach has limitations as the presence of the observer can influence the behavior of the person under observation [45]. To minimize bias, study participants were not informed about the observed hygiene behaviors, though they were generally informed about the purpose of the study. Nonetheless, structured observations remain the gold standard for measuring hygiene behaviors [46,47]. Treatment arms and the control were purposively selected based on the presence of care groups and the CLTS program for the treatment arms rather than random assignment. The control arm was selected based on the absence of care groups and CLTS. Systematic random sampling was used to select study households in each arm.

### Conclusion

We highlight the study design and baseline results from an ongoing BCA trial implemented in rural households in Chiradzulu District, Malawi. The study, implemented within a district-wide WASH program, assesses the effectiveness of CLTS alone against CLTS combined with the CG model for improving sanitation coverage and sustained uptake. The baseline observations indicate a balanced distribution of potential demographic confounders in the trial arms with a slight variation on some WASH proxy measures. We expect to publish the trial findings in peer-reviewed journals in 2025 and share findings at local and national events in Malawi, as well as at international conferences.

## Appendix

Multimedia Appendix 1 CONSORT (Consolidated Standards of Reporting Trials) 2010 checklist.

Multimedia Appendix 2 Survey tool.

Multimedia Appendix 3 Description of study outcomes.

## Abbreviations

CBA: controlled before-and-after
CG: care group
CLTS: community-led total sanitation
DHS: Demographic and Health Survey
HWF: handwashing facility
JMP: Joint Monitoring Programme
MDE: minimum detectable effect
NGO: nongovernmental organization
OD: open defecation
ODF: open defecation free
OR: odds ratio
PCA: principal component analysis
SanQoL-5: Sanitation-related Quality of Life Index
SUN: Scaling Up Nutrition strategy
TA: traditional authorities
W4E: WASH for Everyone
WASH: Water, Sanitation, and Hygiene

## Data Availability

The datasets generated or analyzed during this study are available from the corresponding author on reasonable request.
