# Supplementary material for: Water, Sanitation, and Hygiene for Everyone Intervention Study: Protocol for a Controlled Before-and-After Trial
Source: JMIR Res Protoc. 2025 May 15;14:e68280. doi: 10.2196/68280 (PMC12123240; doi:10.2196/68280)
Supplement: Multimedia Appendix 2 [file resprot_v14i1e68280_app2.docx]

**Household Questionnaire**

| Has consent been given? |
| --- |
| Name of interviewer |
| G1. Name of TA |
| G3. Name of village |
| Scan House ID |
| Is this household under observation? |
| How many people are present at the house household? |
| Enter age of Person present at the household |
| Is there a child less than 5 years old? |
| Who is the person who will be taking care of this child for the next few hours? |
| is this person over 18 years? |
| Is an adult going to be home the entire time of observation? |
|  |
| Observations |
| Event |
| Enter a time |
| Change of observed person |
| Who is being observed now? |
| Reason for ending observation |
| **HAND HYGIENE OPPORTUNITIES** |
| Enter a time |
| Hand hygiene juncture type |
| Specify other |
| How is the child feeding done? |
| hand hygiene behaviour |
| Type of handwashing facility |
| Specify other |
| Handwashing facility location |
| Specify other |
|  |
| Child defacation Observation |
| Enter a time |
| Location of faeces |
| Specify other |
| Faeces disposal |
| Specify other |
| Who disposed of the faeces |
| hand hygiene behaviour |
| Type of handwashing facility |
| Specify |
| Handwashing facility location |
| Specify |
|  |
| 1. How many people live in your household? |
| FOR EACH HOUSEHOLD MEMBER ASK THE FOLLOWING QUESTIONS, STARTING WITH THE RESPONDENT |
| What is the name of Member? |
| What is the age of the member? |
| What is the gender of the member? |
| is this member head of the household? |
| What is the highest grade completed by the member? |
| What is the marital status of the member? |
| Has the member had diarrhoea in the past 7 days? (3 or more loose stools in 24hrs) |
| Has member had a cough or difficulty breathing in the past 7 days? |
| Is there anyone else who doesn't live in the household? For example people who participated in the observation |
| What is their relationship with the household? |
| Does anyone have difficulty seeing, even if wearing glasses? Would you say… |
| Who has difficulty seeing? |
| Does anyone have difficulty hearing, even if using hearing aid(s)? Would you say… |
| Who has difficulty hearing? (select from HHid) |
| Does anyone in this household have difficulty walking or climbing steps? Would you say... |
| Who has difficulty walking or climbing steps? (select from HHid) |
| Does anyone in this household have difficulty remembering or concentrating? Would you say.... |
| Who has difficulty remembering or contrentrating? |
| Does anyone in this household have difficulty with self-care, such as washing all over or dressing? Would you say... |
| Who has difficulty with self care? |
| Using usual language, does anyone in this household have difficulty communicating, for example understanding or being understood? Would you say... |
| Who has difficulty communicating? |
| Is there anyone in this household that has a physical characteristic that makes them different from others in this community, for example albinism? |
| **HOUSEHOLD ASSETS** |
| Does your household have? |
| Electricity [solar or national grid] |
| Radio |
| Television |
| Non-mobile telephone |
| Computer |
| Refrigerator |
| Koloboyi |
| Paraffin lamp |
| Torch |
| Bed with mattress |
| Sofa set |
|  |
| Does any member(s) of this household own: |
| A wrist watch |
| A mobile phone |
| A bicycle |
| A motorcycle or motor scooter |
| An animal-drawn cart |
| A car or truck |
| A boat with a motor |
| Does any member of this household have a bank account? |
| Does your household have a separate room used as a kitchen? |
| How many rooms in your households are used for sleeping? |
| Does this household own any livestock, herds or other farm animals or poultry? |
| Livestock |
| How many of the following do your household own (00 = none; unknown = 999, If 100 or more = 100) |
| Milk cows or bulls |
| Other cattle |
| Donkeys or mules |
| Goats |
| Sheep |
| Pigs |
| Chicken |
| Other poultry |
| Does this household own any agricultural land? |
| How much does this household earn per month? |
| Observe the main material of the exterior walls of the dwelling? |
| Specify other |
| Observe the main materials of the floor of the dwelling |
| Specify other |
| Observe the main materials of the roof of the building |
| Specify other |
| What is the main source of electricity for this household |
| Specify other |
|  |
| **WATER** |
| W1. What is the main source of drinking water for members of this households? |
| Specify other |
| W2. What is the main source of water used by members of this household for cooking and cleaning? |
| Specify other |
| W3. Where is W1/W1A located |
| W4. How long does it take to go To (W1/W1A) there, queue, get water, and return? [Record the total time taken in minutes for a single round trip including queuing] |
| W5. Who usually goes to (W1/W1A) fetch water for your household? |
| W6. Is water always available from (W1/W1A)? |
| W7. What was the (main) reason you were unable to access sufficient quantities of water when needed? |
| Specify other |
| W8. In the past month, for how many days was water from this source [W1/W1a] unavailable when needed? |
| W9. Do you pay to collect water from this source? |
| W10. How frequently do you pay? |
| Specify other |
| W11. How much do you pay? |
| W12. Do members of this households use any source for domestic purposes such as cooking or cleaning? |
| additional water source |
| W13. What is the main _additional_ source of water you use? |
| Specify other |
| W14. Where is W21 located |
| W15. How long does it take to go to (W21) there, queue, get water, and return? |
| W16. Who usually goes to (W21) fetch water for your household? |
| W17.Is water always available from (W21) |
| W18. How many hours per day is water supplied on average |
| W20. Did you collect water for use in your home yesterday? |
| W21. In the last month, has there been any time when your household did not have sufficient quantities of drinking water when needed? |
| W22. Do you have a tank at home that you use to store water? |
| W23. How many litres does this storage container hold? |
| W24. How many times has this tank been filled in the last week? |
| W25. Has there been any time in the last week/month when you have not been able to store sufficient water to meet your needs? |
| W26. Have you or any other household members done anything to this water to make it safer to drink? |
| W27. What do you usually do to the water to make it safer to drink? Anything else? |
| Specify other |
|  |
| **SANITATION ACCESS AND QUALITY** |
| S0. Do you have your own functioning toilet? can you show me |
| S1. What kind of toilet facility do members of your household usually use? |
| Specify other |
| S1. The last time you practised open defecation, how many minutes did it take you to walk to the site, one-way? |
| Sanitation facility |
| S2. Do you share this facility with others who are not members of your household? |
| S3. How many households in total use this toilet facility, including your own household? |
| S4. Do you share this facility only with members of other households that you know or is the facility open to the use of the general public? |
| S5. Where is this toilet facility located? |
| S6. Is your toilet facility easily accessible even to young children, elderly, and disabled people  ? |
| S7. Has your (pit latrine or septic tank) ever been emptied? |
| S8. The last time it was emptied, where were the contents emptied to? |
| Specify other |
| S8b. How many years ago was your latrine pit/septic tank built? |
| S9. How many years ago was your pit latrine/septic tank last emptied? |
| S10. The last time your pit latrine/septic tank {answer to S1} was emptied, who emptied it? |
| Specify other |
| S11. Is everyone in the household able to access and use the toilet at all times of the day and night? |
| S12. What was the (main) reason that household members were unable to use the toilet at all times of the day or night? |
| Specify other |
| S13. Does your sanitation facility leak or overflow wastes at any time of year? |
| S14. Do you know when this sanitation facility was constructed? |
| S15. Who constructed this latrine? |
| Specify |
|  |
| **SANITATION USE** |
| SR1. Does {name_hhid1} usually use this sanitation facility? |
| SR1. Does {name_hhid2} usually use this sanitation facility? |
| SR1. Does {name_hhid3} usually use this sanitation facility? |
| SR1. Does {name_hhid4} usually use this sanitation facility? |
| SR1. Does {name_hhid5} usually use this sanitation facility? |
| SR1. Does {name_hhid6} usually use this sanitation facility? |
| SR1. Does {name_hhid7} usually use this sanitation facility? |
| SR1. Does {name_hhid8} usually use this sanitation facility? |
| SR1. Does {name_hhid9} usually use this sanitation facility? |
| SR1. Does {name_hhid10} usually use this sanitation facility? |
| SR1. Does {name_hhid11} usually use this sanitation facility? |
| SR1. Does {name_hhid12} usually use this sanitation facility? |
| SR1. Does {name_hhid13} usually use this sanitation facility? |
| SR1. Does {name_hhid14} usually use this sanitation facility? |
| SR1. Does {name_hhid15} usually use this sanitation facility? |
| SR1B. The last time {name_hhid1} passed stool, where did she/he go? |
| SR1B. The last time {name_hhid2} passed stool, where did she/he go? |
| SR1B. The last time {name_hhid3} passed stool, where did she/he go? |
| SR1B. The last time {name_hhid4} passed stool, where did she/he go? |
| SR1B. The last time {name_hhid5} passed stool, where did she/he go? |
| SR1B. The last time {name_hhid6} passed stool, where did she/he go? |
| SR1B. The last time {name_hhid7} passed stool, where did she/he go? |
| SR1B. The last time {name_hhid8} passed stool, where did she/he go? |
| SR1B. The last time {name_hhid9} passed stool, where did she/he go? |
| SR1B. The last time {name_hhid10} passed stool, where did she/he go? |
| SR1B. The last time {name_hhid11} passed stool, where did she/he go? |
| SR1B. The last time {name_hhid12} passed stool, where did she/he go? |
| SR1B. The last time {name_hhid13} passed stool, where did she/he go? |
| SR1B. The last time {name_hhid14} passed stool, where did she/he go? |
| SR1B. The last time ${name_hhid15} passed stool, where did she/he go? |
| CF1.  The last time {name_hhid1} passed stool, where did she/he defacate? |
| CF1.  The last time {name_hhid2} passed stool, where did she/he defacate? |
| CF1.  The last time {name_hhid3} passed stool, where did she/he defacate? |
| CF1.  The last time {name_hhid4} passed stool, where did she/he defacate? |
| CF1.  The last time {name_hhid5} passed stool, where did she/he defacate? |
| CF1.  The last time {name_hhid6} passed stool, where did she/he defacate? |
| CF1.  The last time {name_hhid7} passed stool, where did she/he defacate? |
| CF1.  The last time {name_hhid8} passed stool, where did she/he defacate? |
| CF1.  The last time {name_hhid9} passed stool, where did she/he defacate? |
| CF1.  The last time {name_hhid10} passed stool, where did she/he defacate? |
| CF1.  The last time {name_hhid11} passed stool, where did she/he defacate? |
| CF1.  The last time {name_hhid12} passed stool, where did she/he defacate? |
| CF1.  The last time {name_hhid13} passed stool, where did she/he defacate? |
| CF1.  The last time {name_hhid14} passed stool, where did she/he defacate? |
| CF1.  The last time {name_hhid15} passed stool, where did she/he defacate? |
| CF2. The last time {name_hhid1} passed stools, what was done to dispose of the stools? |
| Specify other |
| CF2. The last time {name_hhid2} passed stools, what was done to dispose of the stools? |
| Specify other |
| CF2. The last time {name_hhid3} passed stools, what was done to dispose of the stools? |
| Specify other |
| CF2. The last time {name_hhid4} passed stools, what was done to dispose of the stools? |
| Specify other |
| CF2. The last time {name_hhid5} passed stools, what was done to dispose of the stools? |
| Specify other |
| CF2. The last time {name_hhid6} passed stools, what was done to dispose of the stools? |
| Specify other |
| CF2. The last time {name_hhid7} passed stools, what was done to dispose of the stools? |
| Specify other |
| CF2. The last time {name_hhid8} passed stools, what was done to dispose of the stools? |
| Specify other |
| CF2. The last time {name_hhid9} passed stools, what was done to dispose of the stools? |
| Specify other |
| CF2. The last time {name_hhid10} passed stools, what was done to dispose of the stools? |
| Specify other |
| CF2. The last time {name_hhid11} passed stools, what was done to dispose of the stools? |
| Specify other |
| CF2. The last time {name_hhid12} passed stools, what was done to dispose of the stools? |
| Specify other |
| CF2. The last time {name_hhid13} passed stools, what was done to dispose of the stools? |
| Specify other |
| CF2. The last time {name_hhid14} passed stools, what was done to dispose of the stools? |
| Specify other |
| CF2. The last time {name_hhid15} passed stools, what was done to dispose of the stools? |
| Specify other |
|  |
| **5. HYGIENE** |
| H1. Can you please show me where members of your household most often wash their hands? |
| H2. OBSERVE LOCATION AND RECORD: |
| Specify other |
| H3. Where do you and other members of your household most often wash your hands? |
| Specify other |
| H4. Observe availability of water at the place for handwashing. |
| H5. Observe availability of soap or detergent at the place for handwashing. |
| H6. Observe functionality (all parts are there stick and bottle/state of the container/collection point of water in the ground below water flow) |
| H7. Do you have soap or detergent in your household for washing hands? Can you show it to me? |
| Specify other |
| H8. Record the type of soap observed. Record all that apply. |
| H9. Is there any other soap or detergent in your households that you use for purposes other than washing hands? |
| H10. Record the types of soap observed |
| H11. What do you use this soap for? |
| Specify |
| H12. When do you personally wash your hands with soap? |
| Specify other |
| H13. About how much did your household spend on soap for handwashing and other personal hygiene (e.g. bathing) in the past month? [doesn’t include laundry soap if separate product in this setting] |
| H14. Could you tell me how you use soap when you have soap at this household starting from the most frequent use? [Mark all that apply] |
|  |
|  |
|  |
| 7. IWISE |
| I will now ask you about your experiences with water. For each experience, I want to know how many times this happened to you during the last 4 weeks. |
| IW01. In the last 4 weeks, how often did you worry that you would not have enough water for all of your needs? |
| IW02. In the last 4 weeks, how often did you have to change schedules or plans because of problems with water? |
| IW03. In the last 4 weeks, how often did you not have as much water to drink as you would have liked? |
| IW04. In the last 4 weeks, how often were you not able to wash your hands after dirty activities because of water problems? |
| We would like to know how good or bad your feel your level of water availability is today. This scale is numbered from 0 to 100. 100 means the best level of water availability you can imagine. 0 means the worst level of water availability you can imagine. Please choose a number on the scale to indicate how you feel about availability of water to you today. Consider the quantity and quality of water for all uses, including drinking, washing, cooking, and anything else you consider important |
| Please choose a number on the scale to indicate how you feel about availability of water to you today |
|  |
| 8. SANQOL |
| The following questions are about your sanitation experiences in the past 4 weeks, e.g. defecation, urination, and so on. Please respond with always, sometimes, or never. |
| SQ1. The last time you passed stools, where did you defecate? |
| SQ2. Do you feel disgusted when using the toilet? How often? |
| SQ3. Do you worry that the toilet spreads diseases? How often? |
| SQ4. Do you worry about being seen while using the toilet? How often? |
| SQ5. Do you feel ashamed about using the toilet? How often? |
| SQ6. Do you feel unsafe while using the toilet? How often? |
| SQ7. Do you struggle to access the toilet as quickly as needed? How often? |
| SQ8. Do you feel disgusted when practicing open defecation? How often? |
| SQ9. Do you worry that practicing open defecation spreads diseases? How often? |
| SQ10. Do you worry about being seen while practicing open defecation? How often? |
| SQ11. Do you feel ashamed about practicing open defecation? How often? |
| SQ12. Do you feel unsafe while practicing open defecation? How often? |
| SQ13.Do you struggle to reach somewhere to defecate as quickly as needed? How often? |
| We would like to know how good or bad your feel your level of sanitation is today. This scale is numbered from 0 to 100. 100 means the best sanitation you can imagine. 0 means the worst sanitation you can imagine. Please choose a number on the scale to indicate how you feel about your level of sanitation today. Consider all sanitation practices, including defecation, urination, bathing, menstrual hygiene and any related practices. |
| Please choose a number on the scale to indicate how you feel about your level of sanitation today. |
| Now let’s do an exercise to understand the importance you place on different aspects of sanitation and toilets. Think about sanitation and toilets in general, not your current facility and practices. Consider these 5 cards [enumerator explains each of them]. Please choose the one you consider most important for good quality of life [enumerator places it on ground]. They are all important, but now choose the one you consider least important for good quality of life [enumerator places it on ground below the most important card, and place the other 3 in between]. Now consider these 15 tokens/coins, which are equal in value. I will place one on each card. Please can you allocate the other 10 to represent the relative importance of the cards to you. Please leave at least 1 on each card, even if you think it is not important. |
| Random respondents |
| [enumerator enters number of tokens/coins for “avoid disease”] |
| [enumerator enters number of tokens/coins for “avoid feeling ashamed”] |
| [enumerator enters number of tokens/coins for “avoid feeling disgust”] |
| [enumerator enters number of tokens/coins for “avoid being seen”] |
| [enumerator enters number of tokens/coins for “avoid feeling unsafe”] |
|  |
| HygieneQoL |
| HQ1. Does your body feel more dirty than you would like? How often |
| HQ2. Do you worry that your neighbours think you are not clean? How often? |
| HQ3. Do you find it hard to keep clothes as clean as you would like? How often? |
| HQ4. Do your family members struggle to keep their hands as clean as you would like? How often? |
| We would like to know how good or bad your feel your level of cleanliness is today. This scale is numbered from 0 to 100. 100 means the best cleanliness you can imagine. 0 means the worst cleanliness you can imagine. Please choose a number on the scale to indicate how you feel about your level of cleanliness today. Consider all aspects of cleanliness, including your body, hands, and clothes, and anything else you consider important. |
| Please choose a number on the scale to indicate how you feel about your level of cleanliness today. |
|  |
| 9. SPOT CHECKS |
| SP1. Can I see the toilet facility that you use? |
| SP2. [Direct Observation] What type of sanitation facility is it? |
| SP3. Where does it flush to? |
| SP5. [Direct observation] What type of floor does this latrine have? |
| Specify other |
| SP6. [Direct Observation] What is the condition of the floor? |
| SP7. How clean does the toilet appear? |
| SP8. [Direct observation] Is there a lid/cover for the pit hole? |
| SP9. [Direct observation] How well does this latrine superstructure (walls, door) provide privacy? |
| SP10. [Direct observation] Can the toilet be locked from the inside? |
| SP11. [Direct Observation] Is there evidence that the pit is full or overflowing? |
| SP12. [Direct Observation] Is there evidence of solid waste disposed of in the toilet? |
| SP13. [Direct Observation] Is there enough water present for anal cleasning and toilet flushing? |
| SP14. [Direct Observation] Are anal cleansing materials present inside the toilet?* |
| SP15. [Direct Observation] Which type of anal cleansing materials are present inside the toilet?* |
| Specify other |
| SP16. [Direct Observation] Is the user able to access the latrine right now ( it is not locked, or they have a key) |
| SP17. [Direct Observation] Does the facility show signs of recent use? |
| SP18. [Photo] Take a photo of the inside of the sanitation facility. |
| SP19. [Photo] Take a photo of the outside of the sanitation facility. |
| SP20. OBSERVE, DO NOT ASK - What type of handwashing facility does this household have? |
| Specify other |
| SP21. OBSERVE, DO NOT ASK  - Is there soap or detergent present at the handwashing facility? |
| SP22. OBSERVE DO NOT ASK - Are there any signs of human faeces or dirty diapers on the premises? |
| SP23. OBSERVE DO NOT ASK - Are there any signs of animal faeces on the premises? |
| SP24. Are animals present near or inside the house? |
| SP25. [Direct Observation] Which animals are seen near or inside the house? |
| Specify other |
