# Supplementary material for: Water, Sanitation, and Hygiene for Everyone Intervention Study: Protocol for a Controlled Before-and-After Trial
Source: JMIR Res Protoc. 2025 May 15;14:e68280. doi: 10.2196/68280 (PMC12123240; doi:10.2196/68280)
Supplement: Multimedia Appendix 3 [file resprot_v14i1e68280_app3.docx]

**Appendix 1**. Description of study outcomes.

| **Outcome** | **Description** | **Measurement** | **Unit of measurement** |
| --- | --- | --- | --- |
| **Primary outcome** | | | |
| Sanitation coverage | Access to a private sanitation facility that meets standard definitions of at least unimproved sanitation that respondents self-identify as completed | Self-reported | Household |
| **Secondary outcomes** | | | |
| Basic sanitation coverage | Access to a private sanitation facility that meets JMP standard definitions of at least a basic sanitation facility | Self-reported | Household |
| Sanitation use | Reported location of last defecation event | Self-reported | Individual |
| Sanitation-related quality of life | (SanQoL-5) index is a five-question instrument for measuring the degree of achievement of five attributes (privacy, safety, health, shame and disgust) in a score ranging 0-1. The score is based on the weighted score of responses to five questions on a 3-level frequency scale [32]. | Self-reported | Individual (one respondent per household) |
| Latrine quality | Adapted from methods developed by Tidwell et al[48]. Latrine quality will be a composite measure of observed latrine characteristics, including hygiene, accessibility, sustainability, and observed use. Data will be limited to those households with a latrine at the time of data collection. | Observed | Household |
| Presence of a handwashing facility | Presence of a handwashing where both soap and water are available at the time of data collection | A) Reported or observed  B) Observed only | Household |
| Handwashing behaviour | Observed handwashing opportunities during structured observations and associated hand hygiene action | Observed | Individual |
| Safe disposal of child faeces | Child latrine use or caregiver disposal of child faeces into the latrine for children under five at the time of data collection | Observed | Individual |
